# Randomised controlled trial comparing modified VISTA and gum drop techniques in multiple adjacent gingival recession defects

**DOI:** 10.6026/973206300212161

**Published:** 2025-07-31

**Authors:** Nidhi Prasad, Vidya Sekhar, Sumit Malhotra, Mamta Singh

**Affiliations:** 1 Department of Periodontology and Oral Implantology, ITS dental college, Muradnagar, Ghaziabad Utter Pradesh, India

**Keywords:** Gingival recession defects, gum drop technique (GDT), Modified VISTA, Platelet rich fibrin (PRF)

## Abstract

Gingival recession compromises aesthetics and oral health, with multiple adjacent defects posing a greater clinical challenge. This randomized controlled trial compared the effectiveness of Modified VISTA (m-VISTA) and Gum Drop Technique (GDT) in treating multiple Miller's Class I and II recessions. Thirty-two sites were evaluated for key clinical parameters at baseline and 3 months postoperatively. Both techniques significantly improved gingival parameters, with GDT showing greater reduction in recession depth and gingival thickness. Hence, both m-VISTA and GDT are effective, minimally invasive options, with GDT offering slightly superior clinical gains in the short term.

## Background:

Smile, the simplest form of human communication, is determined by the relationship between the teeth and gingiva. Gingival recession, especially in the anterior region, may lead to an undesirable and unesthetic smile. In a meta-analysis, the overall pooled prevalence of gingival recession was 78.16% and the pooled prevalence of buccal gingival recession was 75.42% [1]. Both non-surgical and surgical interventions have been attempted for the treatment of gingival recession and its associated complications. However, the harmony of a smile cannot be fully restored by non-surgical methods [2]. The gum drop technique (GDT) is a recent soft-tissue grafting procedure that combines minimally invasive incisions with patient-derived blood products (PRF) and minocycline to achieve root coverage [3]. Platelets and leukocytes in A-PRF and i-PRF release growth factors that stimulate neoangiogenesis through vascular endothelial growth factor (VEGF) and fibroblast growth factor [4]. These growth factors also enhance the formation of new stable gingival attachment and support collagen biosynthesis, PDL fibroblast migration, and proliferation, promoting new periodontal ligament (PDL) formation. A contemporary minimally invasive technique, called Modified VISTA (m-VISTA), is used to treat both localized and multiple gingival recession defects [5]. This technique is a variation of the original VISTA (vestibular incision sub periosteal tunnel access) method [6], with the primary difference being the site and type of incisions-a supra-periosteal and sulcular incision are made instead of a sub-periosteal incision. These two minimally invasive techniques have been subjected to limited clinical investigation. Therefore, it is of interest to compare and evaluate the clinical efficacy of Gum Drop Technique (GDT) and Modified VISTA (m-VISTA) in the treatment of multiple adjacent gingival recession defects.

## Materials and Methods:

## Study design:

The present study is a Single center, Randomized controlled clinical trial, conducted on patients who came to the Department of Periodontology and Oral Implantology, Surya Hospital, ITS - CDSR, Ghaziabad, India.

## Study population:

A total of 32 sites of Millers Class I and Class II Gingival Recession defects were selected. The samples were divided into two groups. Group I included 17 sites with Class I and Class II Gingival Recession defects (Miller's) treated with gum drop technique with PRF + root conditioning with Minocycline). Group II included 15 sites with Class I and Class II Gingival Recession defects(Miller's) treated with Modified VISTA (m-VISTA) along with PRF membrane.

## Selection criteria:

Systemically healthy Subjects aged between 18 and 60 years, with multiple adjacent Class I and Class II recession defects (Millers) (≥ 1 mm in depth) both in maxillary and mandibular arch in single rooted teeth, and willing to comply with the study were included in the study. In this study no interproximal bone loss in IOPAR was included. Medically compromised patients, patient's undergone previous periodontal surgery, Recession defects associated with caries, deep abrasion, as well as any pulpal pathology were excluded in the study. Patients who were incompetent for maintenance of oral hygiene were excluded from the study.

## Ethical considerations:

Ethical clearance was obtained from the Institution's Ethical Committee for the study. Ethical principles were adhered to throughout the course of the study. Subjects of the study were selected randomly using envelope method with 1:1 allocation ratio. Blinding was done as two investigators were intended for the study in which one of them is the principal investigator and the other investigator is the outcome assessor.

## Pre-surgical phase:

A detailed dental and medical history of the patients was recorded. Prior to surgery, all patients undergone supragingival scaling and root planning and IOPA were taken in all sites. Patients were advised for blood investigation including complete blood count, Haemoglobin %, platelet count, RBS, bleeding and clotting time, BP. Occlusal stents made of acrylic for positioning the probe and measuring the clinical parameters from a fixed reference point were fabricated for standardization and reproducibility of clinical measurements, with CEJ taken as the reference point.

## Surgical procedure:

All the surgical procedures were performed on an outpatient basis under aseptic conditions. Pre-operative site preparation was done with 5% povidone-iodine solution.

## PRF preparation:

Around 5 ml of whole venous blood was centrifuged at 3000 (rpm) for 10 minutes. PRF was removed and a membrane was obtained from squeezing it between two pieces of moist gauze.

## Group I:

A Gum drop technique was performed. Root conditioning was done using minocycline for 2-3 minutes to remove the smear layer which facilitates the connective tissue attachment. With the help of no. 15 BP blade, small vertical slits was prepared at three spots/points in the alveolar mucosa apical to the mucogingival junction. A tunnel was prepared using a blunt ended tunnelling instrument. The tunnel was extended from the entrance wall of 3 slits sparing till the tip of the Interdental papilla. Patient-derived PRF membranes were introduced into the prepared site. Coronally anchored composite bonded sutures were placed.

## Group II:

The m-VISTA technique was performed. A single vertical incision in the most central section was made and extended to the periosteum and slightly beyond the MGJ. Subsequently intra-crevicular incision was made and extended to at least one of the teeth beyond the treated site till the base of the papilla. Patient-derived PRF membranes were introduced into the prepared site. Coronally anchored composite bonded sutures were placed.

## Post-operative evaluation:

Recession Depth, Width of keratinized gingiva, relative clinical attachment loss, probing depth and gingival thickness was assessed at 3 months after surgery.

## Statistical analysis:

Data collected compiled on to a MS Office excel worksheet & subjected to statistical analysis using SPSS software. Descriptive statistics like frequency (n) & percentage (%) of categorical data, mean & Standard deviation of numerical data at baseline and 3 months recession depth within each group were done using Wilcoxon signed rank test. Intergroup comparison of each clinical parameters value between two groups was done using Mann Whitney test.

## Results:

The gingival recession depth, width of keratinized gingiva, relative clinical attachment level, probing depth, and gingival thickness were recorded for each site in both the groups, at baseline *i.e.* before surgery and 3rd month follow up. The Reduction of gingival recession depth from baseline to third month was higher in group I than group II (p value ≥0.05) (Table 1 - see PDF). Also, from baseline to 3rd month both groups showed statistically significant reduction, with larger differences in group I. (p value ≤ 0.05) (Table 2 - see PDF). In both the groups, the probing depth on all sites did not show significant difference between baseline and third month (p value ≥0.05). Intergroup comparisons of probing depth between two groups, done by Mann Whitney test, revealed non-significant differences between two groups at all time periods (p value ≥0.05) (Table 3 - see PDF). Both groups showed significant reduction in mean relative clinical attachment level from baseline to 3 months, however statistical significance was noted only in group II (p value ≤ 0.05). Inter group comparison in terms of the mean relative clinical attachment level showed no statistically significant difference between the two groups at baseline and 3rd month (p value ≥ 0.05) (Table 4 - see PDF). The mean increase in the mean keratinized gingival width, (baseline to 3 months) was significantly greater in the group I than in the group II (p ≥0.05), but there was no statistically significant difference from baseline and third month between the two groups (p value ≥0.05) (Table 5 - see PDF). The mean increase in the gingival thickness (baseline to 3 months) was significantly greater in the group I than in the group II (p≤0.05). There was no statistically significant difference of gingival thickness at baseline between the groups II, however was not significant in group I (Table 6 - see PDF).

## Discussion:

Multiple root coverage techniques such as coronally advanced flap with or without free mucosal graft, sub epithelial connective tissue graft (SCTG), and guided tissue regeneration are widely recommended for the treatment of gingival recessions [7]. However, these methods often involve extensive incisions and the use of two surgical sites, prompting the development of minimally invasive approaches. In the present study, two such techniques-Gum Drop Technique (GDT) [4] and modified Vestibular Incision Sub periosteal Tunnel Access (m-VISTA) [5] were compared for the treatment of multiple adjacent gingival recessions. The m-VISTA technique is an adaptation of the traditional VISTA method [6], suitable for both localized and multiple recession defects. GDT incorporates minocycline as a root conditioner and platelet-rich fibrin (PRF) to enhance wound healing. The fibrin matrix of PRF offers mechanical adhesion, akin to fibrin glue, stabilizing the flap. Growth factors in PRF further promote angiogenesis and soft tissue regeneration [4]. The findings of this study revealed a statistically significant reduction in mean recession depth from baseline to three months in both the m-VISTA and GDT groups (p < 0.05). These results are consistent with a case report by Elena reporting 90% root coverage using the Gum Drop Technique [8]. The present findings also align with a randomized controlled trial by Zucchelli *et al.* that compared m-VISTA with connective tissue graft (CTG) to coronally advanced flap with CTG [9]. While that study used CTG, the present investigation incorporated PRF in the m-VISTA group. In terms of probing depth, the results are comparable to those reported by Stefan Rebele, where m-VISTA produced no significant probing depth changes over three months [10]. With respect to relative clinical attachment gain, m-VISTA combined with PRF showed superior outcomes compared to the Gum Drop Technique. This aligns with findings from Giovanni Zucchelli, who observed similar clinical attachment gain using m-VISTA and CTG [9, 10- 11]. Additionally, an increase in the width of keratinized gingiva was observed in the m-VISTA group [12]. This increase is likely due to the minimally invasive coronal repositioning technique combined with the biological stimulation provided by PRF [13]. A minor change in gingival thickness was noted in both groups compared to baseline however, the difference was not statistically significant. This is the first study to directly compare the Gum Drop Technique with m-VISTA in managing gingival recession defects. Although both are minimally invasive, they differ in terms of incision placement, use of minocycline for root conditioning, and type of membrane applied. Minocycline enhances collagen production, fibroblast migration, and adhesion by removing the smear layer and conditioning the root surface [14, 15, 16, 17-18]. However, the study's limitations include a small sample size and a limited follow-up period of three months. Future research with larger sample sizes and longer observation periods is essential to evaluate the long-term stability of root coverage, creeping attachment, and changes in the alveolar bone crest.

## Conclusion:

Both Modified VISTA and Gum Drop Technique effectively improved clinical parameters in the treatment of multiple adjacent gingival recessions. GDT demonstrated slightly better outcomes in recession depth reduction and gingival thickness enhancement. Therefore, GDT may offer a preferable minimally invasive alternative for optimal root coverage in suitable cases.
